# Supra-Annular Self-Expanding Versus Intra-Annular Balloon-Expandable Transcatheter Aortic Valve Implantation: A Meta-Analysis of Valve-Related Outcomes

**DOI:** 10.31083/RCM48459

**Published:** 2026-04-21

**Authors:** Mohamed Ali, Trivilou Paraskevi, Muntaser Omari, Mohamed Farag, Michal Kuzemczak, Mohammad Alkhalil

**Affiliations:** ^1^Cardiothoracic Centre, Freeman Hospital, NE7 7DN Newcastle-upon-Tyne, UK; ^2^Division of Emergency Medicine, Poznan University of Medical Sciences, 60-806 Poznan, Poland; ^3^Department of Internal Diseases and Cardiology, Medical University of Warsaw, 02-005 Warsaw, Poland; ^4^Department of Interventional Cardiology and Internal Diseases, Military Institute of Medicine – National Research Institute, 05-119 Legionowo, Poland; ^5^Translational and Clinical Research Institute, Newcastle University, NE4 5PL Newcastle-upon-Tyne, UK

**Keywords:** TAVI, SAPIEN, Evolut, aortic stenosis, meta-analysis, valve thrombosis, HALT

## Abstract

**Background:**

Transcatheter aortic valve implantation (TAVI) is an established treatment for severe aortic stenosis. The two most widely used platforms are either balloon-expandable intra-annular valve (BEV, Edwards) or self-expanding supra-annular valve (SEV) from Medtronic. Comparative data related to clinical and sub-clinical valve thrombosis are limited. The aim of this study-level meta-analysis is to evaluate its incidence and whether this translates into any difference in clinical outcomes.

**Methods:**

Electronic databases were searched from inception through to October 2025 to identify randomised clinical trials of patients receiving either platform. Rates of clinical and subclinical valve thrombosis were identified and compared between the two groups.

**Results:**

In five randomized controlled trials including 1877 patients, the risk of clinical and sub-clinical valve thrombosis was relatively low in both groups. There was a significant 81% reduction in clinical valve thrombosis in patients undergoing SEV compared to BEV [0.4% vs. 2.1%; rate ratio (RR) 0.19, 95% confidence interval (CI) (0.04 to 0.86), *p* = 0.03]. Similarly, the risk of sub-clinical valve thrombosis was significantly lower in the SEV group [0.6% vs. 3.6%; RR 0.22, 95% CI (0.07 to 0.65), *p* = 0.006]. This difference was not translated into increased risk of stroke, valve re-intervention, or death.

**Conclusion:**

Patients undergoing TAVI using SEV compared to BEV have a lower risk of clinical and sub-clinical valve thrombosis in randomized trials, which is largely influenced by small annulus anatomy. Larger studies with longer term follow-up or using a dedicated imaging protocol may provide better insights into the clinical sequelae of this phenomenon.

## 1. Introduction

Transcatheter aortic valve implantation (TAVI) has become a well-established treatment for patients with severe aortic stenosis [1]. Alongside wider adoption, iterative refinements in the design of the transcatheter heart valves (THV) have focused on improving haemodynamic performance, minimising paravalvular regurgitation, and enhancing long-term durability [2, 3, 4, 5].

Nonetheless, the intra-annular balloon expandable valve (BEV) from Edwards Lifesciences and the supra-annular self-expanding valve (SEV) from Medtronic remain the two most widely used platforms. This is likely related to their well-documented evidence against surgical aortic valve replacement (SAVR) [6, 7]. Notably, there are fundamental differences between the two platforms which is reflected in their outcome data. Patients who underwent BEV were less likely to receive a new pacemaker, whilst those with SEV demonstrated better haemodynamic results with lower trans-valvular gradients [6, 7, 8, 9, 10].

Recent data from the Placement of Aortic Transcatheter Valves (PARTNER) 3 trial reporting patient outcomes after 7 years, highlighted an almost five-fold increase in the risk of valve thrombosis in patients who underwent BEV compared to SAVR [6]. In contrast, data from the low risk Evolut study highlighted a comparable risk of valve thrombosis among patients undergoing SEV and SAVR [7]. Valve thrombosis encompasses a spectrum of bioprosthetic valve dysfunctions, ranging from clinically overt thrombosis associated with symptoms or elevated gradients to subclinical phenomena such as hypo-attenuated leaflet thickening (HALT) and reduced leaflet motion, as defined by the Valve Academic Research Consortium (VARC-3) criteria.

Given the continued clinical interest in device-specific outcomes and the importance of tailoring treatment options and optimising prosthesis selection, this study-level meta-analysis is designed to assess the risk of valve thrombosis in patients undergoing BEV and SEV and whether this risk translates into any differences in clinical outcomes, including death and stroke.

## 2. Methods

The MEDLINE, PubMed and Cochrane Central Register databases were searched from their inception through to October 2025 to identify studies comparing clinical outcomes among patients undergoing BEV with Sapien from Edwards Lifesciences and SEV using Core valve/Evolut from Medtronic. The search strategy used the following keywords: aortic valve stenosis, severe aortic stenosis, Edwards Sapien, Sapien 3, Sapien XT, Medtronic Evolut, core valve, Evolut R, Evolut Pro, TAVI, TAVR, valve thrombosis, clinical valve thrombosis, and sub-clinical valve thrombosis.

Studies were eligible if they met the following criteria: (i) randomised comparative design; (ii) included adult patients undergoing TAVI for severe aortic stenosis; (iii) compared BEV using Edwards Sapien versus Medtronic Core valve/ Evolut valves; (iv) and reported at least one of the predefined outcomes of interest. This includes outcomes such as death, stroke, and clinical or sub-clinical valve thrombosis. Observational studies, case reports, editorials, reviews, conference abstracts, registry-only analyses, and studies without a direct comparator were excluded.

All the included articles were assessed by two authors (MK, MAlk) using the prespecified inclusion criteria described above. None of the authors was an investigator in any of the selected studies and any disagreement about including any study was resolved by consensus. Study title and abstract content for each study was screened during the initial search results, and relevant studies were retrieved for a full review. Subsequently, full study reports were assessed to confirm whether they met the inclusion and exclusion criteria to be synthesised in the present meta-analysis. Previously published systematic reviews and meta-analyses on similar topics were reviewed to cross-check the results. To ensure proper evaluation and adequate inclusion of the studies, the Preferred Reporting Items for Systematic Reviews and Meta-Analyses (PRISMA) guidelines were followed during the search strategy for identifying relevant records [11]. Patients in the BEV group were considered the control group, while those in the SEV group were defined as the experimental group.

Data were extracted using a standardised form and included variables such as year of publication, sample size, patient demographics, follow-up duration, and outcomes of interest. Where multiple publications reported outcomes from the same study cohort, the most recent or most complete dataset was used to avoid duplication. This was applied to the SMall Annuli Randomized To Evolut™ or SAPIEN™ Trial (SMART) whereby the two year outcome data was presented at the American College of Cardiology conference but only the one-year outcome was published [8].

The main outcomes of this study-level meta-analysis included clinical and subclinical valve thrombosis, valve reintervention, and stroke. Other variables such as death, pacemaker rate, and moderate or severe paravalvular regurgitation were also recorded.

### Statistical Analysis

As reported in the included studies, continuous data are presented as mean ± SD or as median (range), while categorical variables are presented as percentages. Using study-level data, the difference in the treatment effect was reported using the rate ratio (RR) with 95% confidence intervals (CI) (adjusted by person-years to account for potential differences in the included studies’ follow-up). To calculate the pooled RRs, a random-effects model was used to relatively weigh the studies equally since all included studies were randomized trials (the results were consistent when applying a fixed effect). Publication bias was evaluated using a funnel plot and statistical heterogeneity was assessed with the I^2^ statistic. The statistical analysis was performed using RevMan software version 5.4 (Cochrane Informatics & Technology, London, UK), and *p* < 0.05 was considered statistically significant.

## 3. Results 

**Fig. 1.**
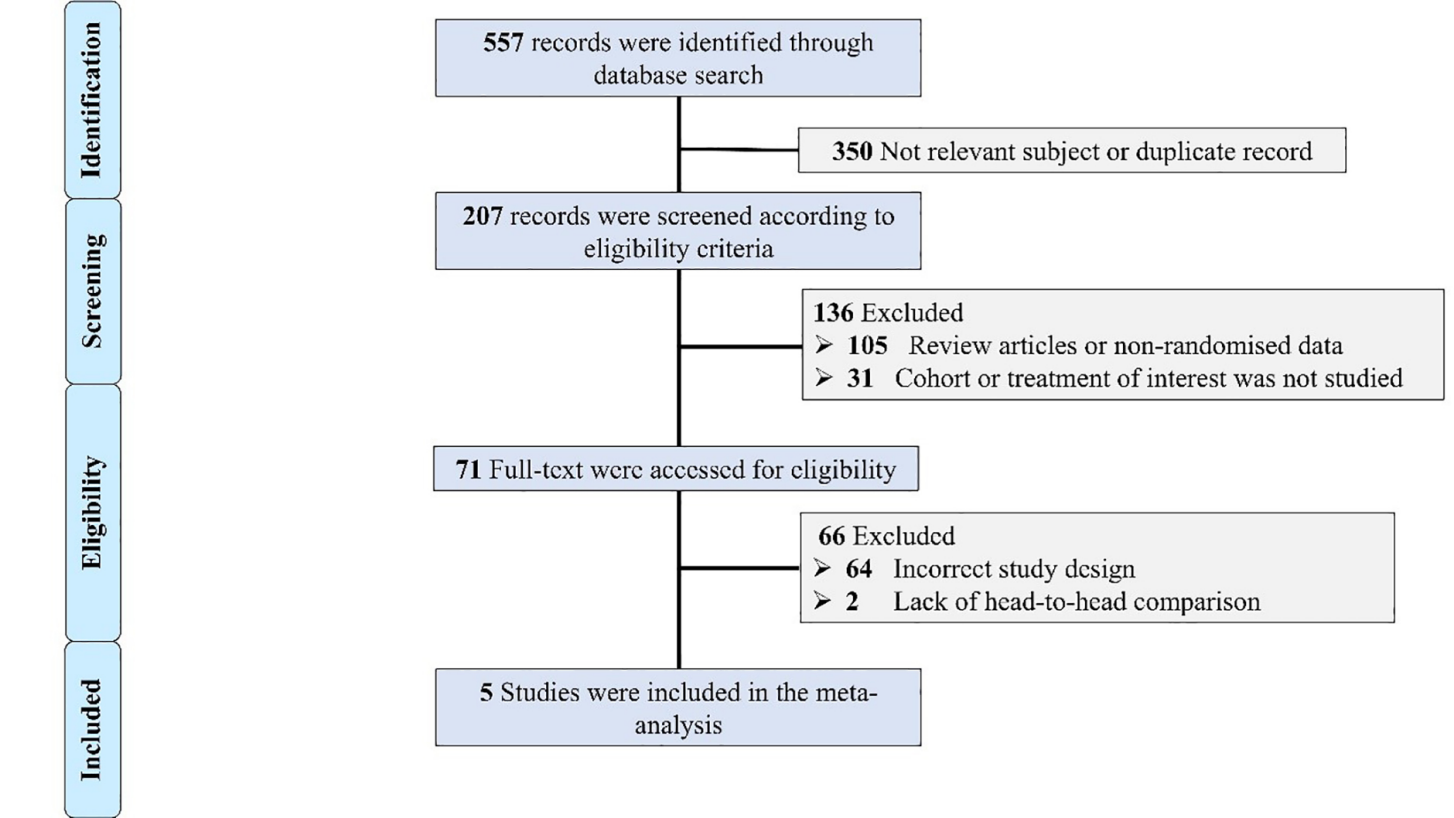
**Flow chart of the included studies**. Selection process of identified randomized trials evaluating the outcomes of patients with severe aortic stenosis undergoing trans-catheter aortic valve implantation (TAVI) using supra-annular self-expanding versus intra-annular balloon-expandable valve (BEV).

The used search strategy yielded 557 records that were initially screened. The flow chart of screening and including studies is presented in Fig. 1. Five randomized trials comparing BEV versus SEV in patients with severe symptomatic aortic stenosis undergoing TAVI were included [8, 12, 13, 14, 15].

The total number of included patients was 1877, 939 (50%) of which underwent TAVI with BEV compared to 938 (50%) who underwent SEV. Baseline clinical characteristics are presented in Table 1 (Ref. [8, 12, 13, 14, 15]). All the included studies recruited patients who are relatively old (average age around 80 years) with a relatively high proportion of female patients.

**Table 1.** **Baseline characteristics of the included studies**.

Study	Number of patients	Age	Male (%)	Society of thoracic surgeons	Primary endpoint	Duration of follow up
Abdel-Wahab* et al*. [12] (2020)	241	80	36	5.9	Device success, which is a composite endpoint including successful vascular access and deployment of the device and retrieval of the delivery system, correct position of the device, intended performance of the heart valve without moderate or severe regurgitation, and only 1 valve implanted in the proper anatomical location.	5 years
Nuche *et al*. [13] (2023)	98	79	53	5.4	The rate of severe PPM or moderate-severe aortic regurgitation at 30 days.	12 months
Royen *et al*. [15] (2025)	384	80	54	2.6	Composite of all-cause mortality, all stroke, bleeding (Valve Academic Research Consortium (VARC) types 3 and 4), acute kidney injury (stages 2, 3, and 4), major vascular complications, moderate or severe prosthetic valve regurgitation, and conduction system disturbances resulting in a new permanent pacemaker implantation (PPI) as per VARC-3.	12 months
Feistritzer *et al*. [14] (2025)	438	81	49	4.0	Composite of all-cause mortality, stroke, moderate or severe PVL, and permanent pacemaker implantation at 30-day follow-up.	5 years
Herrmann *et al*. [8] (2024)	716	80	13	3.3	Composite of death, disabling stroke, or rehospitalization for heart failure.	24 months

**Fig. 2.**
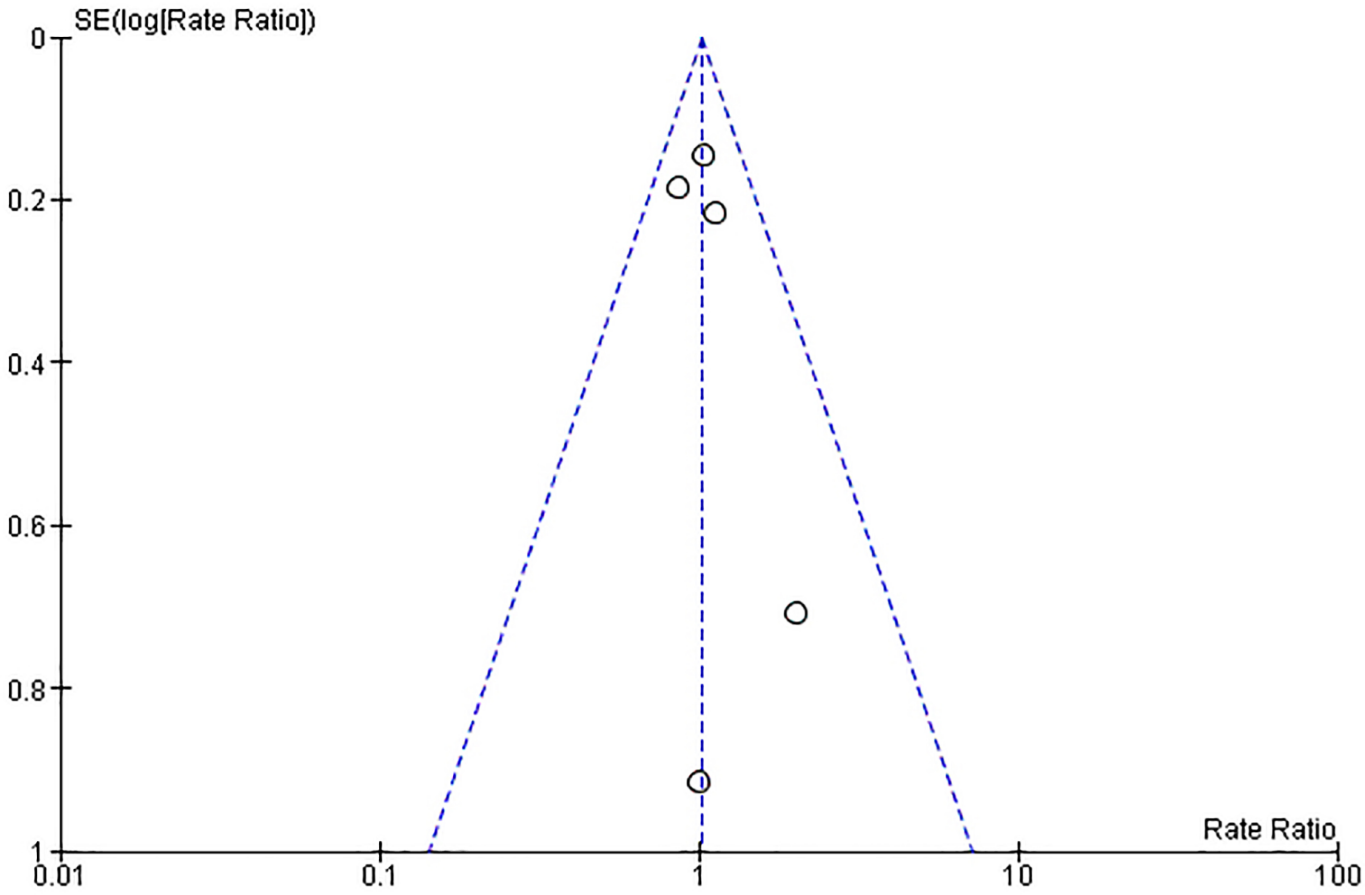
**Funnel plot of the included studies**. The horizontal axis represents the rate ratio (RR), while the vertical axis reflects the standard error of log RR. The vertical and sloping dotted lines represent the pooled RR and expected 95% confidence intervals (CIs) for a given standard error (SE), respectively.

In total, there were 408 (21.7%) deaths, 124 (6.6%) strokes, 343 (18.3%) pacemakers, and 138 (7.4%) patients with moderate to severe aortic regurgitation. Risk of publication bias was evaluated by visual assessment of the funnel plot shown in Fig. 2. There was moderate between-trial heterogeneity (*I*^2^ = 50%); however, all included studies were randomized clinical trials and their data were reported according to intention-to-treat analysis.

**Fig. 3.**
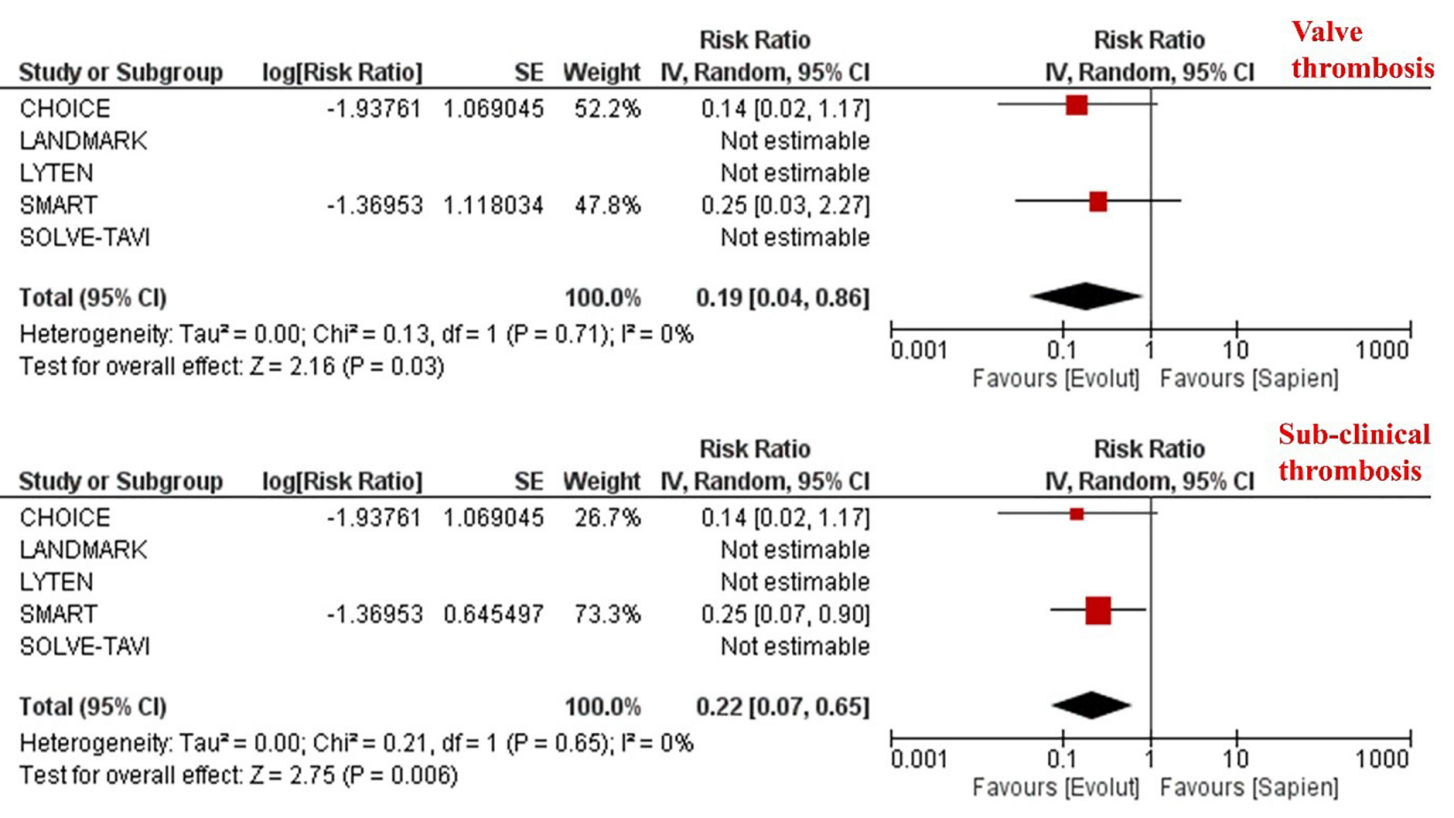
**Meta-analysis of clinical and sub-clinical valve thrombosis according to the trans-catheter heart valve platform**. Individual and pooled rate ratios of clinical and sub-clinical valve thrombosis with 95% confidence intervals of patients undergoing supra-annular self-expanding versus intra-annular balloon-expandable valves.

**Fig. 4.**
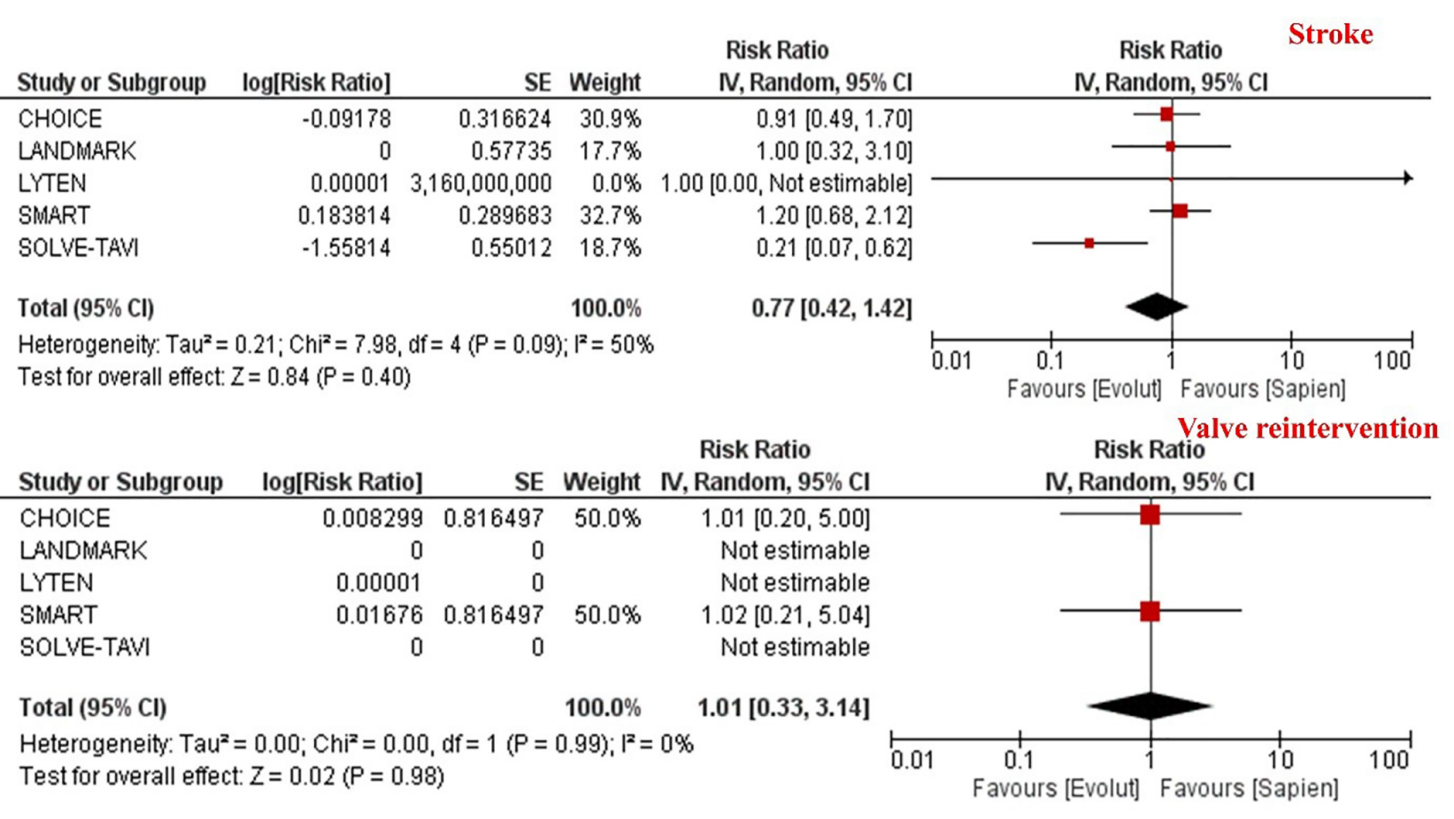
**Meta-analysis of stroke and valve reintervention according to the trans-catheter heart valve platform**. Individual and pooled rate ratios of stroke and valve reintervention with 95% confidence intervals of patients undergoing supra-annular self-expanding versus intra-annular balloon-expandable valves.

The overall incidence of valve thrombosis as defined by each study’s protocol was 1.2% and was significantly lower in patients undergoing TAVI with SEV compared to BEV [0.4% vs. 2.1%; RR 0.19, 95% CI (0.04–0.86), *p* = 0.03] (Table 2, Fig. 3). Similarly, the incidence of sub-clinical valve thrombosis was 1.82% and was significantly lower in patients receiving TAVI with SEV compared to BEV [0.6% vs. 3.6%; RR 0.22, 95% CI (0.07–0.65), *p* = 0.006] (Table 2, Fig. 3). However, this was not translated into a statistically significant difference in risk of stroke which was comparable between the two groups [6.0% vs. 7.2%; RR 0.77, 95% CI (0.42–1.42), *p* = 0.40] (Table 2, Fig. 4). Likewise, there was no difference in the incidence of valve reintervention between patients receiving SEV compared to BEV [0.6% vs. 0.6%; RR 1.01, 95% CI (0.33–3.14), *p* = 0.98] (Table 2, Fig. 4). Given the low event rates of valve reintervention, the results were recalculated using Peto Odds Ratio and there was no difference in the incidence of valve reintervention between the two platforms [1.01; 95% CI (0.32, 3.17), *p* = 0.98]

**Table 2.** **Clinical outcomes of patients with severe aortic stenosis undergoing trans-catheter aortic valve implantation (TAVI) with supra-annular self-expanding versus intra-annular balloon expandable valve**.

	Supra-annular self-expanding	Intra-annular balloon-expandable	Rate ratio (95% confidence interval)	*p* value
Valve thrombosis	2 (0.2%)	11 (1.2%)	0.19 (0.04–0.86)	0.03
Sub-clinical valve thrombosis	4 (0.4%)	19 (2.0%)	0.22 (0.07–0.65)	0.006
Stroke	56 (6.0%)	68 (7.2%)	0.77 (0.42–1.42)	0.40
Valve reintervention	6 (0.6%)	6 (0.6%)	1.01 (0.33–3.14)	0.98
Death	204 (21.7%)	204 (21.7%)	1.01 (0.83–1.23)	0.92
Pacemaker	191 (20.4%)	152 (16.2%)	1.26 (1.02–1.56)	0.03
Aortic regurgitation (more than mild)	73 (7.8%)	65 (6.9%)	1.54 (0.66–3.61)	0.32

There was no difference in death [21.7% vs. 21.7%, RR 1.01 95% CI (0.83–1.23), *p* = 0.92], or more than mild aortic regurgitation [7.8% vs. 6.9%; RR 1.54, 95% CI (0.66–3.61), *p* = 0.32] (Table 2). The risk of requiring a permanent pacemaker was significantly higher in patients receiving SEV compared to BEV [20.4% vs. 16.2%, RR 1.26; 95% CI (1.02–1.56), *p* = 0.03] (Table 2).

## 4. Discussion

The main findings of this study-level meta-analysis can be summarised as follows: (1) the risk of clinical or sub-clinical valve thrombosis was low in patients with severe aortic stenosis undergoing TAVI; (2) there was a significant 80% reduction in the risk of clinical or sub-clinical valve thrombosis in patients undergoing supra-annular self-expanding compared to an intra-annular balloon-expandable valve; however, (3) this risk was not translated into any difference in stroke, valve reintervention or death.

Recent studies highlighted comparable clinical outcomes in patients with severe aortic stenosis undergoing TAVI compared to SAVR who are at low surgical risk [6, 7]. This was reflected in the recent guidelines whereby the cut-off age for patients to be considered for TAVI was reduced from 75 to 70 years [1]. The life expectancy for this new group of patients (i.e., between 70 and 74 years) is 12 years and efforts need to be focused to ensure that the durability of THV would at least match this timeline [16, 17]. Therefore, structural valve deterioration becomes increasingly important when discussing treatment options for patients with severe aortic stenosis.

Valve thrombosis is one type of bioprosthetic valve dysfunction according to VARC-3 criteria [18]. Previous studies have focused on understanding its prevalence, mechanisms, prognosis and potential treatment options [19, 20, 21, 22, 23]. Several anatomical and THV-related factors have been linked to the development of valve thrombosis [19, 24]. From a mechanistic standpoint, differences in neosinus geometry, leaflet position, and flow washout between supra-annular and intra-annular valve designs may influence local flow stasis and thrombogenicity. These findings have important implications for future transcatheter valve design, emphasizing the importance of optimizing leaflet kinematics and sinus washout to mitigate thrombotic risk [24].

Other clinical factors such as body mass index, inflammatory status, and prothrombotic conditions have also been considered as part of the pathophysiology of valve thrombosis [24]. Overall, valve thrombosis is a multifactorial phenomenon. Whilst valve platform represents one contributory mechanism among many, including patient anatomy, other factors such as anticoagulation strategy, hemodynamics, and procedural techniques are very relevant to the development of valve thrombosis.

The incidence of clinical valve thrombosis is relatively low and has been reported to be less than 1% in previous studies [25]. The current study-level meta-analysis, which only included data from large randomized studies, corroborated this finding. Patients with clinical valve thrombosis can present with heart failure symptoms, or thromboembolic events. Elevated trans-valvular gradients can also be associated with clinical or sub-clinical valve thrombosis, although previous data have not been consistent in supporting this finding [26, 27]. On the other hand, the risk of HALT has been reported to be relatively high and up to 52% in some series [28]. Additionally, the HALT phenomenon was demonstrated to be dynamic in nature with resolution of some cases and development of new cases between 30 days and one year [20, 26]. Although subclinical valve thrombosis and HALT have attracted increasing attention, their clinical significance remains uncertain. To date, most studies—including the present analysis—have failed to demonstrate a consistent association between HALT and hard clinical endpoints such as stroke, mortality, or valve reintervention. HALT should currently be regarded as an imaging biomarker reflecting altered leaflet–flow interaction and provides physicians with an opportunity to address this phenomenon using oral anticoagulation. Importantly, routine anticoagulation in patients undergoing TAVI was associated with a higher risk of death and bleeding compared to anti-platelet strategies [29]. Therefore, a tailored approach, factoring in the risk of HALT should be considered when approaching patients undergoing TAVI. Both PARTNER and low risk Evolut studies reported an incidence in almost one third of cases [20, 26]. Importantly, these studies had dedicated imaging protocol using computed tomography (CT) to evaluate the risk of HALT. This may explain the lower reported incidence in our study which highlighted outcomes according to clinically indicated imaging tests.

The current study highlighted higher risk of clinical and subclinical valve thrombosis in patients undergoing BEV versus SEV. A previous meta-analysis of 25 studies including more than 11,000 patients demonstrated that patients undergoing TAVI with intra-annular compared to supra-annular valves had a two-fold increased risk of sub-clinical valve thrombosis [30]. Whether the trapped native aortic leaflets in close proximity to the THV leaflets play a role in promoting thrombus formation has not been fully elucidated [24]. On the other hand, the presence of THV thrombosis has been linked to flow stasis in the native sinus and neosinus [31]. Blood stagnation related to slow wash out and reduced velocities can potentially promote platelet activation and the development of thrombus formation [24].

In contrast to Bogyi *et al*. [30], our meta-analysis did not link the increased risk of valve thrombosis in patients undergoing BEV compared to SEV with clinical outcomes such as stroke or valve reintervention. The difference between the designs of both studies may explain the discordant results. Our study only included large, randomized trials and, therefore, baseline characteristics are balanced, and the role of other competing risks is likely to be minimized. Additionally, valve thrombosis is considered a rare event and large observational studies with long-term outcomes are more likely to capture such events. Therefore, assessing the risk of rare events would require large real-world data that was excluded from our analysis.

The management of clinical or sub-clinical valve thrombosis remains focused on oral anticoagulation. When compared to antiplatelet agents, oral anticoagulation was associated with a significant reduction in the risk of sub-clinical valve thrombosis [23]. However, oral anticoagulation following TAVI was associated with increased mortality and bleeding, challenging its routine use [29]. Similarly, dual antiplatelet treatment was associated with a higher risk of bleeding compared to a single antiplatelet strategy, with no difference in the risk of sub-clinical valve thrombosis between the two strategies [24, 32].

Our study has several limitations that need to be highlighted. The reported analysis included study-level and not individual-level data and, therefore, assessment of clinical or sub-clinical valve thrombosis according to certain anatomical features, such as small annuli, was not possible. Additionally, the duration of follow-up varies within the included studies and the incidence of sub-clinical valve thrombosis is known to be dynamic and change over time. Finally, the definition of clinical or sub-clinical valve thrombosis was according to the criteria used by each individual study and was not standardized in the current meta-analysis. Furthermore, these events were detected in some studies based on clinically indicated imaging rather than systematic CT protocols. In fact, some of the included studies did not report any thrombosis events and the main conclusions were derived from two relatively large studies, adding more challenges to the interpretation of the results.

## 5. Conclusion 

Patients undergoing TAVI using SEV compared to BEV have a lower risk of clinical and sub-clinical valve thrombosis in randomized trials largely influenced by small annulus anatomy. Larger studies with longer-term follow-up or using a dedicated imaging protocol may provide better insights into the clinical sequelae of this phenomenon.

## Availability of Data and Materials

Data are available from the corresponding author on a reasonable request.

## Supplementary Material

Supplementary material associated with this article can be found, in the online version, at https://doi.org/10.31083/RCM48459.

## References

[1] Praz F, Lanz J, Adamo M, Borger M (2025). 2025 ESC/EACTS Guidelines for the management of valvular heart disease. *European Journal of Cardio-thoracic Surgery: Official Journal of the European Association for Cardio-thoracic Surgery*.

[2] Stinis CT, Abbas AE, Teirstein P, Makkar RR, Chung CJ, Iyer V (2024). Real-World Outcomes for the Fifth-Generation Balloon Expandable Transcatheter Heart Valve in the United States. *JACC. Cardiovascular Interventions*.

[3] Gada H, Khalil RF, Chetcuti SJ, Deeb GM, Grubb KJ, Greenbaum AB (2025). 30-Day and 1-Year Outcomes From the Optimize PRO TAVR Evolut FX Addendum Study. *JACC. Cardiovascular Interventions*.

[4] Worthley SG, Giordano A, Corcione N, Nombela-Franco L, De Marco F, Walton A (2025). 30-Day and 1-Year Outcomes of Navitor Transcatheter Aortic Valve in Low- or Intermediate-Risk Patients. *JACC. Cardiovascular Interventions*.

[5] Antonio Baz J, Burgdorf C, Frerker C, Cruz González I, Antoni Gômez J, Graham J (2025). First-In-Human Experience of the New Fully Repositionable IMPERIA Delivery System to Implant the ALLEGRA Transcatheter Heart Valve in Patients With Severe Calcific Aortic Stenosis or Degenerated Surgical Bioprosthesis: Thirty-Day Results of the EMPIRE I Study. *Structural Heart: the Journal of the Heart Team*.

[6] Leon MB, Mack MJ, Pibarot P, Hahn RT, Thourani VH, Kodali SH (2025). Transcatheter or Surgical Aortic-Valve Replacement in Low-Risk Patients at 7 Years. *The New England Journal of Medicine*.

[7] Forrest JK, Yakubov SJ, Deeb GM, Gada H, Mumtaz MA, Ramlawi B (2025). 5-Year Outcomes After Transcatheter or Surgical Aortic Valve Replacement in Low-Risk Patients With Aortic Stenosis. *Journal of the American College of Cardiology*.

[8] Herrmann HC, Mehran R, Blackman DJ, Bailey S, Möllmann H, Abdel-Wahab M (2024). Self-Expanding or Balloon-Expandable TAVR in Patients with a Small Aortic Annulus. *The New England Journal of Medicine*.

[9] Omari M, Durrani T, Diaz Nuila ME, Thompson A, Irvine T, Edwards R (2025). Cardiac output in patients with small annuli undergoing transcatheter aortic valve implantation with self-expanding versus balloon expandable valve (COPS-TAVI). *Cardiovascular Revascularization Medicine: Including Molecular Interventions*.

[10] Abdalwahab A, Omari M, Alkhalil M (2025). Aortic Valve Intervention in Patients with Aortic Stenosis and Small Annulus. *Reviews in Cardiovascular Medicine*.

[11] Page MJ, McKenzie JE, Bossuyt PM, Boutron I, Hoffmann TC, Mulrow CD (2021). The PRISMA 2020 statement: an updated guideline for reporting systematic reviews. *BMJ (Clinical Research Ed.)*.

[12] Abdel-Wahab M, Landt M, Neumann FJ, Massberg S, Frerker C, Kurz T (2020). 5-Year Outcomes After TAVR With Balloon-Expandable Versus Self-Expanding Valves: Results From the CHOICE Randomized Clinical Trial. *JACC. Cardiovascular Interventions*.

[13] Nuche J, Abbas AE, Serra V, Vilalta V, Nombela-Franco L, Regueiro A (2023). Balloon- vs Self-Expanding Transcatheter Valves for Failed Small Surgical Aortic Bioprostheses: 1-Year Results of the LYTEN Trial. *JACC. Cardiovascular Interventions*.

[14] Feistritzer HJ, Kurz T, Vonthein R, Schröder L, Stachel G, Eitel I (2025). Effect of Valve Type and Anesthesia Strategy for TAVR: 5-Year Results of the SOLVE-TAVI Trial. *Journal of the American College of Cardiology*.

[15] Royen NV, Amat-Santos IJ, Hudec M, Bunc M, Ijsselmuiden A, Laanmets P (2025). Early outcomes of the novel Myval THV series compared to SAPIEN THV series and Evolut THV series in individuals with severe aortic stenosis. *EuroIntervention: Journal of EuroPCR in Collaboration with the Working Group on Interventional Cardiology of the European Society of Cardiology*.

[16] Martinsson A, Nielsen SJ, Milojevic M, Redfors B, Omerovic E, Tønnessen T (2021). Life Expectancy After Surgical Aortic Valve Replacement. *Journal of the American College of Cardiology*.

[17] Foroutan F, Guyatt GH, O’Brien K, Bain E, Stein M, Bhagra S (2016). Prognosis after surgical replacement with a bioprosthetic aortic valve in patients with severe symptomatic aortic stenosis: systematic review of observational studies. *BMJ (Clinical Research Ed.)*.

[18] VARC-3 WRITING COMMITTEE, Généreux P, Piazza N, Alu MC, Nazif T, Hahn RT (2021). Valve Academic Research Consortium 3: updated endpoint definitions for aortic valve clinical research. *European Heart Journal*.

[19] Hatoum H, Gooden SCM, Sathananthan J, Sellers S, Kutting M, Marx P (2021). Neosinus and Sinus Flow After Self-Expanding and Balloon-Expandable Transcatheter Aortic Valve Replacement. *JACC. Cardiovascular Interventions*.

[20] Makkar RR, Blanke P, Leipsic J, Thourani V, Chakravarty T, Brown D (2020). Subclinical Leaflet Thrombosis in Transcatheter and Surgical Bioprosthetic Valves: PARTNER 3 Cardiac Computed Tomography Substudy. *Journal of the American College of Cardiology*.

[21] Chakravarty T, Søndergaard L, Friedman J, De Backer O, Berman D, Kofoed KF (2017). Subclinical leaflet thrombosis in surgical and transcatheter bioprosthetic aortic valves: an observational study. *Lancet (London, England)*.

[22] Waksman R, Bhogal S, Gordon P, Ehsan A, Wilson SR, Levitt R (2023). Transcatheter Aortic Valve Replacement and Impact of Subclinical Leaflet Thrombosis in Low-Risk Patients: LRT Trial 4-Year Outcomes. *Circulation. Cardiovascular Interventions*.

[23] De Backer O, Dangas GD, Jilaihawi H, Leipsic JA, Terkelsen CJ, Makkar R (2020). Reduced Leaflet Motion after Transcatheter Aortic-Valve Replacement. *The New England Journal of Medicine*.

[24] Marchandot B, Trimaille A, Kikuchi S, Truong DP, Carmona A, Morel O (2025). Subclinical Leaflet Thrombosis and Subclinical Aortic Valve Complex Thrombosis in TAVR. *JACC. Advances*.

[25] Latib A, Naganuma T, Abdel-Wahab M, Danenberg H, Cota L, Barbanti M (2015). Treatment and clinical outcomes of transcatheter heart valve thrombosis. *Circulation. Cardiovascular Interventions*.

[26] Blanke P, Leipsic JA, Popma JJ, Yakubov SJ, Deeb GM, Gada H (2020). Bioprosthetic Aortic Valve Leaflet Thickening in the Evolut Low Risk Sub-Study. *Journal of the American College of Cardiology*.

[27] Hein M, Schoechlin S, Schulz U, Minners J, Breitbart P, Lehane C (2022). Long-Term Follow-Up of Hypoattenuated Leaflet Thickening After Transcatheter Aortic Valve Replacement. *JACC. Cardiovascular Interventions*.

[28] Fujita K, Matsumura K, Sugimoto K, Onishi K, Kakehi K, Yoshida A (2025). Early clinical outcomes of transcatheter aortic valve implantation using the NAVITOR system. *Cardiovascular Intervention and Therapeutics*.

[29] Dangas GD, Tijssen JGP, Wöhrle J, Søndergaard L, Gilard M, Möllmann H (2020). A Controlled Trial of Rivaroxaban after Transcatheter Aortic-Valve Replacement. *The New England Journal of Medicine*.

[30] Bogyi M, Schernthaner RE, Loewe C, Gager GM, Dizdarevic AM, Kronberger C (2021). Subclinical Leaflet Thrombosis After Transcatheter Aortic Valve Replacement: A Meta-Analysis. *JACC. Cardiovascular Interventions*.

[31] Vahidkhah K, Barakat M, Abbasi M, Javani S, Azadani PN, Tandar A (2017). Valve thrombosis following transcatheter aortic valve replacement: significance of blood stasis on the leaflets. *European Journal of Cardio-thoracic Surgery: Official Journal of the European Association for Cardio-thoracic Surgery*.

[32] Alkhalil M, Edwards R, Puri R, Kalra A, Zaman A, Das R (2022). Aspirin Versus Dual Antiplatelet Therapy in Patients Undergoing Trans-Catheter Aortic Valve Implantation, Updated Meta-Analysis. *Cardiovascular Drugs and Therapy*.

